# Family functioning and health-related quality of life of inpatients with coronary heart disease: a cross-sectional study in Lanzhou city, China

**DOI:** 10.1186/s12872-022-02844-x

**Published:** 2022-09-06

**Authors:** Hongchen Zhang, Yanhong Wang, Xiaoqing Cai, Nan Tang, Siqi Wei, Yanwei Yang

**Affiliations:** 1 School of Nursing, Lanzhou University, Lanzhou, 730000 Gansu China; 2 Department of Cardiology, The 940th Hospital of Joint Logistic Support Force of PLA, Lanzhou, 730050 Gansu China; 3 Department of Stomatology, The 940th Hospital of Joint Logistic Support Force of PLA, No. 333 South Binhe Road, Qilihe District, Lanzhou, 730050 Gansu China

**Keywords:** Coronary heart disease, Health-related quality of life, Sickness impact profile, Family functioning, Family APGAR index, Northwest China

## Abstract

**Background:**

A key outcome in coronary heart disease (CHD) is Health Related Quality of Life (HRQoL), and family functioning is important in the management of CHD. But few studies have examined both together, and little is known about them among inpatients with CHD in less developed areas of China. Therefore, this study aimed to assess the HRQoL and family functioning status of inpatients with CHD in Lanzhou from Northwest China, and identify the factors that affect their HRQoL.

**Methods:**

A cross‑sectional study was conducted in 224 CHD inpatients at one major hospital. Sociodemographic data and disease information of CHD inpatients were collected by face-to-face using a structured questionnaire and data were also obtained from patient medical records. HRQoL was measured using the Sickness Impact Profile (SIP). Family functioning was measured using the family APGAR index. Multiple binary logistic regression analysis (MBLRA) was used to explore potential risk factors associated with HRQoL, and Pearson’s correlations were used to assess the relationship between family functioning and HRQoL.

**Results:**

The overall, physical and psychosocial SIP scores were 25.03 ± 8.52, 18.61 ± 9.90 and 28.08 ± 9.64, respectively. The total family APGAR score was 6.11 ± 2.45. MBLRA found older age, poorer cardiac function and more severe disease were associated with poorer HRQoL, while better family functioning, higher monthly income, and urban living were associated with better HRQoL. Family functioning was weakly to moderately correlated with total and psychosocial HRQoL.

**Conclusions:**

Older and less affluent inpatients with lower educational level, less family support and more severe CHD have poorest quality of life, and health care providers should consider interventions to support them.

**Supplementary Information:**

The online version contains supplementary material available at 10.1186/s12872-022-02844-x.

## Background

Coronary heart disease (CHD) is the most prevalent type of cardiovascular disease, which nowadays has been the second largest cause of death for Chinese adults [1]. Due to improved treatment and management of CHD, the number of CHD survivors in low- and middle-income countries has increased, with an impact on morbidity and mortality [2, 3]. In China, the mortality attributable to CHD was 17.06 million in 2018 [4]. Globally, according to the current statistics, CHD accounted for more than 10% of health care costs, and almost 50% of the global health care costs for CHD occurred in Asia, which is continuously increasing [5]. In terms of disease management, the maintenance of CHD patients’ health-related quality of life (HRQoL) is more important than survival time [6].

Treatment, overall, aims to improve patients’ HRQoL. HRQoL not only is a predictor of general wellbeing, but is also a strong indicator of CHD patients’ health status, making HRQoL measurement useful for assessing the socio‑economic impact, burden of illness, effectiveness of interventions and treatments among patients after a cardiac event [7, 8]. In CHD patients, HRQoL is related to numerous variables, including gender and other socioeconomic factors, personality and neurotic axis disorders such as stress, depression and anxiety. Also, behavior change variables include smoking cessation, medication adherence, self-care, sedentary lifestyle, controlled cholesterol, blood pressure and weight loss [9, 10]. In China, there have only been two studies, which used somewhat different methodologies, were conducted in different areas of China, and gave somewhat different results [7, 11]. One study used the European Quality of Life 5-dimensions (EQ-5D) scale and EQ Visual Analog Scale (EQ-VAS) to measure the HRQoL of CHD patients in rural communities of Fangshan district in Beijing in Northern China. It found that multiple factors independently predicted these two HRQoL measures, including marital status, physical activity, alcohol drinking, family size and comorbid stroke. Comorbid diabetes mellitus additionally predicted EQ-VAS scores, but not EQ-5D. The other study used the 36-item Short-Form to measure HRQoL in outpatients with newly diagnosed stable angina in Chongqing, Southwest China. Physical and mental HRQoL were positively correlated with exercise and monthly income and negatively associated with age [11]. Whereas now, this study is the first to examine the HRQoL of CHD inpatients in Northwest China.

Social support, generally from the family, has an important influence on HRQoL in CHD patients, probably due to stress reduction and practical assistance [12, 13]. The relationship between social support and HRQoL is particularly marked for older adults, and those with effective family functioning displayed better physical and mental HRQoL [14, 15]. However, few studies have examined the family functioning status of CHD patients and the association between their family functioning and HRQoL [7, 11]. Here, it is assumed that families with better functioning will be more capable of offering good support for patients with CHD.

We positioned our study on a sample of CHD inpatients in Lanzhou, Northwest China. Firstly, this study focused on CHD inpatients in Northwest China for the first time, where the economy is underdeveloped and most patients have lower education and income levels. Secondly, CHD inpatients can reflect the health problems and the factors affecting the HRQoL in a concentrated and comprehensive way, because they are always under acute pressure. And lastly, inpatients were recruited because data collection was simple, reliable and cost-efficient [8, 16, 17].

Therefore, the current study aimed to assess the HRQoL and family functioning status of inpatients with CHD in Lanzhou from Northwest China, and identify the factors that affect their HRQoL.

## Methods

### Study design

**Fig. 1:**
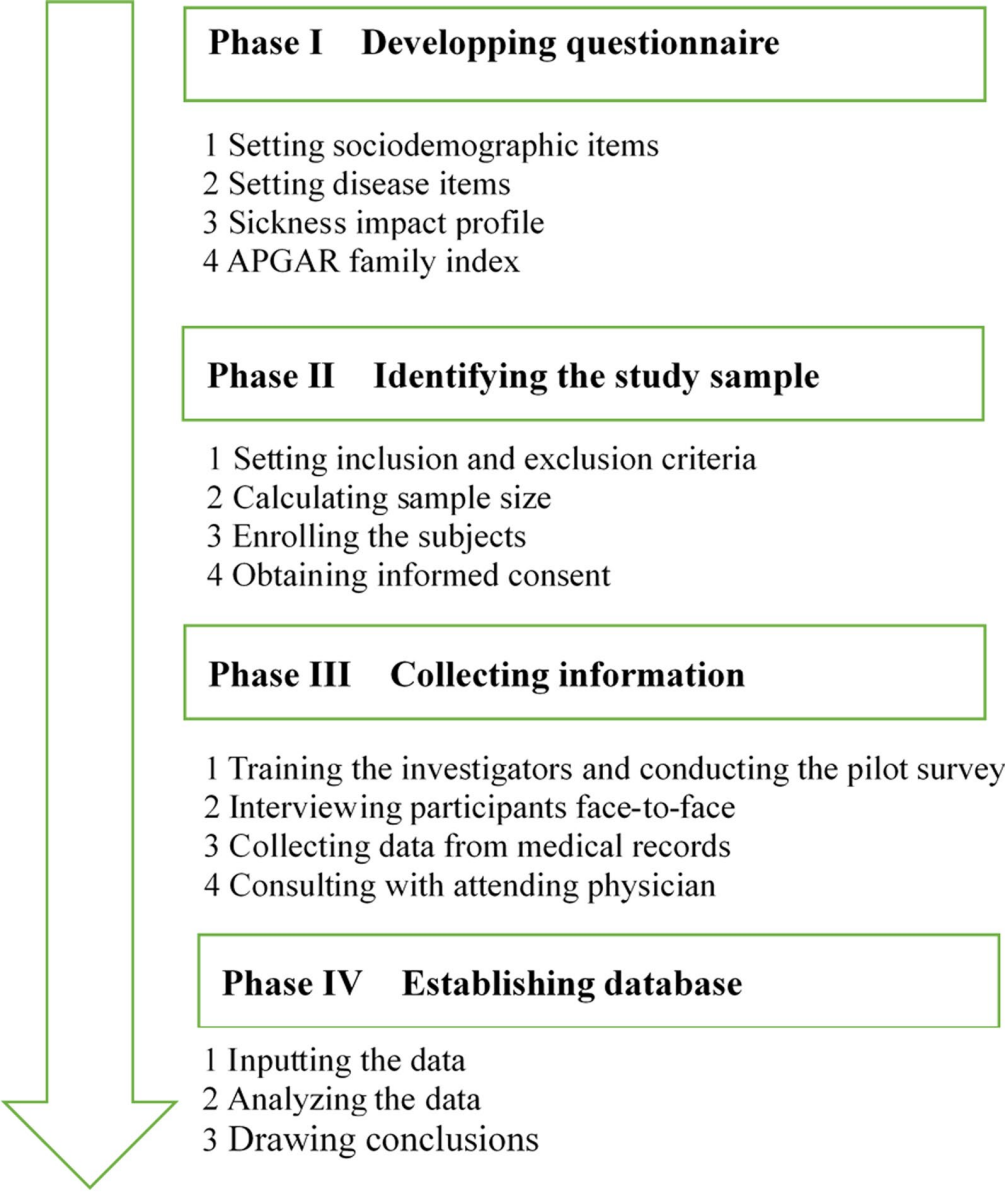
The flow chart of this study

A cross-sectional survey ran from November 2017 to June 2018 in the Department of Cardiology at the 940th Hospital of Joint Logistics Support Force, Lanzhou, Gansu Province, Northwest China. We recruited inpatients who were admitted for cardiac imaging, intervention, or surgery for CHD. Inpatients meeting the inclusion criteria were successively included in the study. Data were collected by several investigators who had been trained to conduct face-to-face interviews with participants to ensure reliability and validation. We performed a pilot study on 15 inpatients to test the feasibility and clarity of the questions. The inpatients participating in the pilot study were not included in the final analysis. The flow chart of this study was shown in Fig. 1.

This work was approved by the Ethics Committee of School of Nursing, Lanzhou University in China (No. hlxy20170016) and all methods were performed in accordance with the Declaration of Helsinki and other relevant guidelines and regulations. Written informed consent was signed by each participant prior to data collection.

### Inclusion and exclusion criteria

The inclusion criteria [7, 8] were inpatients who: (1) were local residents aged 18 years or older living locally for at least 5 years; (2) were diagnosed with CHD according to the criteria of the International Society of Cardiology and World Health Organization [2]; (3) had not experienced a surgical procedure during this hospitalization; (4) had normal thinking and communication ability assessed at interview; (5) agreed to participate in the study (the refusal rate was 7.4% (n = 18)). Exclusion criteria were the following: (1) known cardiac diseases other than CHD; (2) having acute medical conditions; (3) known neuropathy disorders and syndromes affecting cognitive abilities [8, 11].

### Sample size

In the current study, 224 CHD inpatients were included, surpassing the minimum sample size needed of 115, derived with following formula: *n* = (*Z*_1−*α*/2_/*δ*)^2^ × *p* × (1−*p*). Where N is the sample size, Z is the statistic corresponding to the confidence level, *p* is the expected prevalence, and δ is the tolerance error. Assuming, *α* = 0.05, *Z*_1−*α*/2_ = 1.96, *δ* = 3%, and we chose *p* from a recent Chinese study with CHD prevalence rate of 2.78% [6]. In addition, another 5 to 10% was added in an attempt to minimise errors and increase the study’s reliability as much as possible.

### Participants’ sociodemographic and disease data

The following sociodemographic characteristics were collected and categorized by a structured questionnaire: gender (male or female), age (< 60, 60–70, or > 70 years), marital status (married or unmarried, including single, divorced or widowed), educational level (≤ junior high school, senior high school or secondary technical school, or ≥ college degree), monthly income (< 1000, 1000–3000, or > 3000 Yuan), residence (countryside or city). Clinical data of participants, including illness duration, cardiac function grade, CHD classification and other data, were obtained from their attending physician and medical records. Once data were collected, they were anonymized for analysis.

### HRQoL, sickness impact profile

HRQoL was assessed with the Sickness Impact Profile (SIP) through personal interview. The SIP scale is a comprehensive and objective tool to evaluate HRQoL with any chronic disease, which can avoid response bias and own a good reliability and validity [18–24]. It has been used in patients with cirrhosis, amyotrophic lateral sclerosis, narcolepsy and others [22–24]. In 1990, Chinese scholars Li Junrong and Zhang Xinping translated it into Chinese, which has been widely used in patients with chronic obstructive pulmonary disease, colorectal cancer, stroke and other conditions in China [25, 26]. The empirical studies show that it is an effective functioning status measuring instrument [18, 19]. Therefore, this study used the SIP scale to assess CHD inpatients for the first time. This version of SIP includes 136 items grouped into 12 categories. Here the following category scores were used. Total score, two summary scores—Physical score (body care and movements, mobility, and ambulation), Psychosocial score (emotional behavior, social interactions, alertness, and communication)—plus five individual scores; work, sleep and rest, eating, home management, and recreation and pastime. Because the respondents in this study were mostly retired, the Work category of SIP was not used. SIP scores are expressed as percentages of disease effect, with higher scores indicating a worse HRQoL. The Cronbach's α coefficient of this sample was 0.84 and those of subscales ranged from 0.80 to 0.89.

### Family functioning, APGAR

The Family APGAR index is a five-item (adaptation, partnership, growth, affection, and resolution) scale used to evaluate perceived family functioning with a three-point option of each item ranging from 0 (hardly ever) to 2 (almost always), and it is suitable for use in any ages [27]. The final Family APGAR score is the sum of all 5 items ranging from 0 to 10, for it a total APGAR score of 0–3 is poor, 4–6 is fair, and 7–10 suggests good family functioning [28, 29]. The Chinese version was developed in 1992, and the simplicity and convenience of the scale have made it widely used in family functioning assessment of various patient groups in China [30]. It is reliable and has good test–retest validity, with the correlation coefficient of 0.80–0.83 [28, 31].The Cronbach's α coefficient of this sample was 0.81.

### Statistical analyses

Data analysis used SPSS 17.0 (SPSS Inc., Chicago, IL, USA). Two-sample t tests, or one-way analysis of variance was performed in the univariate analysis as appropriate, and multiple binary logistic regression analysis (MBLRA) was performed to identify risk factors influencing HRQoL, using statistically significant variables from univariate analyses as independent variables and the three aggregated SIP scores (total, physical and psychosocial SIP scores) as dependent variables. We also calculated Pearson’s correlations to assess correlations between HRQoL scores and family support scores. Continuous variables are shown as means with standard deviations, and categorical variables are shown as percentages. Dependent variable in MBLRA must be dichotomous variable, so overall, physical and psychosocial SIP scores must be transformed. Thus, we chose the 25th percentile (P_25_) values of SIP scores as cut-off points because P_25_ of the distribution is considered to be the most appropriate dichotomous indicator of health concepts [16, 32]. Odds ratios (ORs) and 95% confidence intervals (CIs) were calculated in MBLRA. Statistical significance was set at *P* < 0.05.

## Results

### Basic characteristics of participants

Basic participant characteristics are shown in Table 1. There were approximately even proportions of men and women. Age ranged from 34 to 85 years (mean 65.72 ± 10.38 years). Most were married, most had not completed school beyond Junior high, and most lived in the city. Most had grade II or III cardiac function and most had angina pectoris.

**Table 1:** Socio-demographic and clinical characteristics of the study sample (N = 224)

Variable	Frequency (n)	%
Gender		
Male	113	50.4
Female	111	49.6
Age (years)		
< 60	69	30.8
60–70	78	34.8
> 70	77	34.4
Marital status		
Married	187	83.5
Unmarried (single, divorced or widowed)	37	16.5
Educational level		
≤ Junior high school	150	67.0
Senior high school or secondary technical school	47	21.0
≥ College degree	27	12.0
Monthly income (Yuan)		
< 1000	52	23.2
1000–3000	100	44.7
> 3000	72	32.1
Residence		
Countryside	56	25.0
City	168	75.0
Illness duration (year)		
< 1	73	32.6
1–5	75	33.5
5–10	42	18.8
> 10	34	15.2
Cardiac function grade		
I	7	3.1
II	128	57.2
III	82	36.6
IV	7	3.1
Disease classification		
Angina pectoris	190	84.8
Ischemic cardiomyopathy	22	9.8
Myocardial infarction	12	5.4

### SIP scores

The total SIP score was 25.03 ± 8.52. The HRQoL sequence of 2 SIP dimensions and 4 independent categories, from high to low, were as following: eating (12.99 ± 5.33), physical dimension (18.61 ± 9.90), which included mobility (28.24 ± 13.07), ambulation (21.75 ± 10.89), and body care and movement (13.83 ± 10.53); psychosocial dimension (28.08 ± 9.64), which included emotional behavior (48.84 ± 19.59), social interaction (29.98 ± 12.49), communication (7.25 ± 8.74), and alertness behavior (25.11 ± 13.95); home management (29.93 ± 16.43), sleep and rest (32.29 ± 14.45), and recreation and pastimes (56.62 ± 14.67). So, recreation and pastimes functioning had the largest impairment of more than 50%.

### Perceived family functioning (APGAR scores) of the CHD inpatients

The mean total Family APGAR score was 6.11 ± 2.45. Among these inpatients, 119 (53.1%) perceived good family functioning, 63 (28.1%) perceived fair family functioning, and 42 (18.8%) perceived poor family functioning. Five aspect scores of Family APGAR scale were listed as following: Adaptation (1.44 ± 0.67), Partnership (1.33 ± 0.68), Growth (1.52 ± 0.63), Affection (1.09 ± 0.67), and Resolution (0.72 ± 0.57).

### Correlations between HRQoL (SIP scores) and family functioning (Family APGAR scores) in CHD inpatients

Here, correlation coefficients of 0–0.3 are considered nonexistent correlations; 0.3–0.49 low; 0.5–0.69 moderate; 0.7–0.89 high; and 0.9–1.00 very high [33]. As shown in Table 2, all APGAR subscales were related to Psychosocial SIP, most to Total SIP and none to Physical SIP. APGAR total score was most strongly correlated with Psychosocial SIP.

**Table 2:** Correlations between HRQoL (SIP scores) and family functioning (Family APGAR scores) in CHD inpatients

Family APGAR scores	HRQoL (SIP scores)		
	Total SIP	Physical SIP	Psychosocial SIP
Adaptability	− 0.168*	0.094	− 0.403***
Partnership	− 0.355***	− 0.132	− 0.501***
Growth	− 0.266**	− 0.039	− 0.411***
Affection	− 0.326***	− 0.034	− 0.559***
Resolve	− 0.303***	− 0.099	− 0.455***
Total APGAR score	− 0.370***	− 0.051	− 0.611***

Values in this table are correlation coefficients. The bold values indicate meaningful correlations

*P < 0.05; **P < 0.01; ***P < 0.001

### The effects of different study variables on SIP scores in univariate analyses

Relationships between study variables and the SIP scores of CHD inpatients are shown in Table 3. All had significant relationships to SIP scores, except gender was unrelated to the psychosocial dimension.

**Table 3:** Relationships between study variables and HRQoL (SIP scores) of CHD inpatients

Variable	SIP scores (mean ± SD)					
	Physical SIP	*P*	Psychosocial SIP	*P*	Total SIP	*P*
Gender						
Male	16.50 ± 8.43	0.001**	27.48 ± 9.97	0.347	23.49 ± 8.20	0.006**
Female	20.75 ± 10.82		28.69 ± 9.30		26.60 ± 8.59	
Age						
< 60	11.21 ± 6.08	< 0.001***	22.55 ± 7.91	< 0.001**	18.50 ± 6.13	< 0.001***
60–70	17.69 ± 5.92		29.37 ± 9.69		25.05 ± 6.77	
> 70	26.00 ± 10.70		31.54 ± 7.92		30.69 ± 7.92	
Marital status						
Married	17.60 ± 9.20	0.001**	27.43 ± 9.44	0.024*	24.16 ± 8.29	< 0.001***
Unmarried	23.70 ± 11.71		31.35 ± 10.11		29.44 ± 8.38	
Educational level						
≤ Junior high school	20.74 ± 10.00	< 0.001***	30.27 ± 9.16	< 0.001***	27.09 ± 8.09	< 0.001***
Senior high school or secondary technical school	15.50 ± 7.60		25.36 ± 9.09		22.31 ± 7.40	
≥ College degree	12.15 ± 8.84		20.64 ± 8.50		18.33 ± 8.12	
Monthly income (Yuan)						
< 1000	21.78 ± 10.76	0.002**	33.19 ± 9.48	< 0.001***	28.79 ± 8.54	< 0.001***
1000–3000	19.18 ± 9.71		28.62 ± 8.80		25.81 ± 8.00	
> 3000	15.51 ± 8.69		23.62 ± 8.93		21.23 ± 7.78	
Residence						
Countryside	19.21 ± 10.55	0.044*	28.70 ± 10.43	0.039*	25.30 ± 8.88	0.028*
City	14.34 ± 9.62		20.80 ± 9.30		18.91 ± 8.38	
Illness duration (year)						
< 1	14.63 ± 8.70	< 0.001***	25.10 ± 8.95	0.001**	21.47 ± 7.46	< 0.001***
1–5	18.55 ± 9.97		28.11 ± 9.19		24.92 ± 8.58	
5–10	21.04 ± 9.67		29.48 ± 10.49		27.18 ± 8.72	
> 10	24.26 ± 9.05		32.65 ± 9.14		30.25 ± 6.89	
Cardiac function grade						
I	11.88 ± 7.66	< 0.001***	19.83 ± 7.00	< 0.001***	16.84 ± 6.35	< 0.001***
II	14.37 ± 8.27		24.89 ± 8.69		21.32 ± 7.40	
III	24.38 ± 7.74		32.88 ± 8.65		30.36 ± 6.08	
IV	35.18 ± 10.61		38.27 ± 8.07		38.62 ± 4.67	
Disease classification						
Angina pectoris	16.90 ± 8.65	< 0.001***	27.06 ± 9.61	0.001**	23.71 ± 9.61	< 0.001***
Ischemic cardiomyopathy	27.30 ± 9.39		34.93 ± 6.66		32.96 ± 5.52	
Myocardial infarction	29.68 ± 14.00		31.55 ± 9.47		31.33 ± 10.88	
Level of family support						
Good (APGAR score = 7–10)	18.80 ± 11.21	< 0.001***	23.82 ± 7.89	< 0.001***	23.13 ± 8.85	< 0.001***
Fair (APGAR score = 4–6)	21.09 ± 8.62		29.13 ± 7.69		25.30 ± 7.59	
Poor (APGAR score = 0–3)	25.85 ± 7.64		38.57 ± 8.37		30.00 ± 6.83	

*P < 0.05; **P < 0.01; ***P < 0.001

### MBLRA for risk factors associated with SIP in CHD inpatients

The results of MBLRA for risk factors associated with SIP are shown in Tables 4, 5, 6, respectively. The P_25_ values of total, physical and psychosocial SIP scores as cut-off points were 18.240, 11.363 and 20.974, respectively. The results in Table 4 showed that total SIP was negatively associated with patients’ older age (OR = 1.274), poorer cardiac function grade (OR = 38.624) and more serious disease classification (OR = 28.984), but positively associated with residence in city (OR = 0.204) and better family support (OR = 0.575).

**Table 4:** MBLRA for risk factors associated with HRQoL measured by total SIP in CHD inpatients

Variable	B	S.E	Wald	*P*	OR	95% CI
Age	0.242	0.048	24.902	< 0.001	1.274	1.158–1.401
Residence place	− 1.588	0.725	4.793	0.029	0.204	0.049–0.847
Cardiac function grade	3.654	1.130	10.453	0.001	38.624	4.216–196.852
Disease classification	4.686	1.098	8.263	0.008	28. 984	3.343–213.948
Family functioning	− 0.553	0.185	8.931	0.003	0.575	0.400–0.827

**Table 5:** MBLRA for risk factors associated with HRQoL measured by physical SIP in CHD inpatients

Variable	B	S.E	Wald	*P*	OR	95% CI
Age	0.206	0.045	20.593	< 0.001	1.228	1.124–1.342
Cardiac function grade	3.277	1.083	9.150	0.002	26.492	3.170–221.437
Family functioning	− 0.996	0.239	17.315	< 0.001	0.369	0.231–0.590

**Table 6:** MBLRA for risk factors associated with HRQoL measured by psychosocial SIP in CHD inpatients

Variable	B	S.E	Wald	*P*	OR	95% CI
Age	0.115	0.029	16.051	< 0.001	1.122	1.061–1.188
Educational level	− 0.645	0.315	4.201	0.040	0.525	0.283 − 0.972
Monthly income	− 0.825	0.408	4.208	0.038	0.592	0.266–0.946
Family functioning	− 0.754	0.162	21.779	< 0.001	0.471	0.343–0.646

The results in Table 5 showed that physical HRQoL was negatively related to patients’ older age (OR = 1.228) and poorer cardiac function grade (OR = 26.492), but positively related to better family support (OR = 0.369).

As shown in Table 6, patients’ older age (OR = 1.122) was negatively associated with psychosocial HRQoL, but higher educational level and monthly income (OR = 0.525, 0.592) and better family support (OR = 0.471) were positively associated with psychosocial SIP.

## Discussion

The present study aimed to assess the family functioning and HRQoL among CHD inpatients from Northwest China. It found that older patients with lower incomes, lower educational level and less perceived family functioning had poorer psychosocial quality of life. Not surprisingly, there was also evidence that additionally the severity of the coronary disease affected physical quality of life. Overall quality of life was furthermore affected by disease classification and reduced by living rurally. However, rural living generally tends to reduce quality of life for everyone [1, 6]. Recreations and pastimes were the aspect of quality of life that was most affected by CHD.

The effects of age are consistent with those of previous studies [7, 11, 17, 34]. These could be due to a general reduction in HRQoL with age, or to the increased duration of CHD [9]. Anxiety and depression scores and disease severity increase with age, which would lead to a reduction in physical and mental abilities, resulting in reduced HRQoL [35]. Longer disease course, poor prognosis and the incurability of CHD may worsen the psychological burden for elderly inpatients, which may also reduce HRQoL [36].

Family functioning was a key protective factor for HRQoL, similar to previous studies [14, 15]. Better family functioning is an important influence on the development and prognosis of CHD and on HRQoL in CHD patients [12]. While dysfunctional families may also offer support, especially practical support, clinincal experience suggests that this will be stressful for the patients with CHD. Here, most inpatients had fair (29.9%) to good (53.1%) family functioning. This may be due to the Chinese traditional family values and lifestyle, making families more functional and more capable of offering support. For example, inpatients can get financial support from their family members, which meets the most basic needs for their treatment and rehabilitation [37]. Moreover, the family members also can give the inpatients basic daily care and spiritual consolation, motivating inpatients to cope positively with the disease and eventually improving their health status [38].

Not surprisingly, in logistic regression, cardiac function grade was a major risk factor for total and physical HRQoL in CHD inpatients, with odds ratios of 38.624 and 26.492, respectively. Poorer cardiac functioning reduces activity, increases mortality and reduces effective cardiac rehabilitation [10, 39]. Compared with inpatients with angina pectoris, inpatients with more severe CHD, such as myocardial infarction and ischemic cardiomyopathy, have more serious signs and symptoms and poorer cardiac function, leading to lower total HRQoL. More severe disease typically has a great impact on inpatients’ physical activity ability, which is most noticeable in inpatients with myocardial infarction, which can require initial complete bed rest for 2–12 days, depending on the inpatients’ individual condition, until the condition is stable. Moreover, inpatients with myocardial infarction score more highly on depression and anxiety than inpatients with other CHD [40], which are associated with worsened physical, mental, and social HRQoL [17, 41, 42].

Respondents living rurally had lower HRQoL than those in the city. One study in Israel of people with CHD found no urban–rural difference [8], however, there may be substantial differences between the natures of rural and urban life in Israel and Northwest China. Through the interviews, we found that the majority of inpatients living rurally complained that they were unable to afford their medical expenses, and many risk factors for CHD, such as fatigue, were unavoidable. In addition, they did not obtain timely diagnosis and medication adjustment, therefore suffered from a greater overall health dysfunction than did urban inpatients.

Higher monthly income was protective for psychosocial HRQoL, with an OR value of 0.592, as found previously [7, 11, 43, 44]. Inpatients with lower income may have a limited ability to obtain effective treatments, care and rehabilitation to improve their clinical outcomes, ultimately resulting in poor HRQoL. Higher educational level was also protective for psychosocial HRQoL, with an OR value of 0.525. 88% of CHD inpatients do not have college degrees in present study, because Lanzhou is a relatively backwards area with a weak economic and health care environment. Lower educational level is often related to poorer disease cognitions, health concepts and lifestyle, which lead to lower HRQoL [45].

Here, women had poorer HRQoL in univariate analysis, which is consistent with some studies [46, 47] but inconsistent with others [7, 8]. However, gender was not a significant predictor in multivariate analyses, which is consistent with some studies [7, 8, 11] but inconsistent with others [17, 47]. Nor was marital status related to HRQoL, again consistent with two studies [8, 11] but inconsistent with another [7]. This contradiction may be due to different samples, settings and different HRQoL scales used in different studies.

Medical staff should develop policies and interventions to improve the HRQoL of CHD inpatients in Northwest China, such as improving the medical insurance system to reduce the economic burden, and strengthening the disease knowledge education to enhance patients’ understanding of CHD. Particular attention should be paid to the older inpatients with lower income, lower educational level and living in rural areas, who were found to exhibit poorer HRQoL. In addition, interventions should be adopted to improve family functioning that is effective to promote their HRQoL. For example, patients should strengthen communication with family members. Meanwhile, family members should actively provide emotional and information support for patients.

### Limitations

Several limitations can be noted in the present study. Firstly, data were collected from CHD inpatients at one hospital in one city, which may cause a sampling bias and may affect the representativeness of the study. Secondly, our study focused on hospitalized patients, which may present a higher rate of reduced HRQoL compared to outpatients. And the exclusion of some inpatients with CHD also could cause biases. Finally, the study focused on family functioning and some demographic factors, so there is a need for further studies of other determinants of HRQoL in Chinese CHD populations. However, the study is the first to focus on the association between HRQoL and family functioning of CHD inpatients in China.

## Conclusions

Older inpatients with lower incomes, lower educational level and less family functioning had poorer psychosocial quality of life. There was also evidence that additionally the severity of cardiac functioning affected physical quality of life. Overall quality of life was furthermore affected by disease classification and reduced by living rurally. Improving the family functioning of CHD inpatients is an effective way to promote their HRQoL.

## Supplementary Information

**Additional file 1**. The questionnaire of this study.

**Additional file 2**. The database of this study.

## Data Availability

All data generated or analysed during this study are included in this published article and its Additional files 1, 2.

## Abbreviations

CHD: Coronary Heart Disease
SIP: Sickness Impact Profile
APGAR: Adaption, Partnership, Growth, Affection, and Resolve
MLRA: Multivariate Logistic Regression Analysis
WHO: World Health Organization
PLA: Chinese People's Liberation Army
SD: Standard deviation
ANOVA: Analysis of variance
ORs: Odds ratios
