# Supplementary material for: Family functioning and health-related quality of life of inpatients with coronary heart disease: a cross-sectional study in Lanzhou city, China
Source: BMC Cardiovasc Disord. 2022 Sep 6;22:397. doi: 10.1186/s12872-022-02844-x (PMC9446873; doi:10.1186/s12872-022-02844-x)
Supplement: Supplementary file 1 — Additional file 1. The questionnaire of this study. [file 12872_2022_2844_MOESM1_ESM.docx]

**Questionnaire of Family functioning and other factors influencing health-related quality of life in patients with coronary heart disease in Lanzhou**

Date: SN：

The impact of coronary heart disease (CHD) on your health has always been the focus of medical staff.  The purpose of this survey is to understand the changes of CHD on your health, a total of 12 aspects of a comprehensive evaluation of the disease on your functional status, as well as family care, in order to further carry out CHD health education, rehabilitation nursing, promote your health to provide a scientific basis.  This questionnaire is anonymous, to ensure the confidentiality of the information you fill in, and ensure that the information obtained is only used for research, please feel free to fill in truthfully, sincerely thank you for your assistance!

**PART 1：Personal and Medical Condition (Tick or fill in the blank according to your actual situation)**

1.Gender：A. Male B. Female

2. Date of birth：

3.Marital Status：A. Married B. Unmarried（Single、Divorced、Widowed）

4.Professional：A. Leader B. Worker C. Farmer D. Office clerk E. Others（ ）

5.Educational level：A. ≤ Junior high school B. Senior high school or secondary technical school

C. ≥ College degree

6. Religious Faith：A. Yes B. No

7. Monthly income (Yuan)：A.＜1000 B. 1000-3000 C. ＞3000

8.Payment method of expenses：A. All reimbursement B. Partly reimbursement （Percentage： ） C. All pay own way

9. Residence：A. Countryside B. City

10. **Be filled in by medical staff**

Height：（ ） Weight：（ ） BP：（ ）

Blood Fat：A. Normal B. Abnormal Blood Glucose：A. Normal B. Abnormal

Left ventricle Ejection Fraction(LEF)：（ ） Cardiac Function Grade（ ）

Disease Classification: A. Angina pectoris B. Ischemic cardiomyopathy C. Myocardial infarction

Number of Heart Stent（ ）

**PART2：Sickness Impact Profile (The items are all the experiences in your life. Due to your sickness, your activities have changed compared with usual. Please tick "√" in the corresponding box according to the actual situation to choose whether or not. )**

**Ⅰ— Sleep & Rest**

1. Lie down and rest almost all day. YES□ NO□

2. Sitting almost all day. YES□ NO□3. Sleeping or napping for most of the day and night. YES□ NO□4. Lie down and rest several times during the day. YES□ NO□

5. Often sitting half asleep. YES□ NO□6. Poor sleep, such as waking up early, often awake at night, difficult to sleep for a long time.

YES□ NO□7. Often sleep or nap during the day. YES□ NO□

**Ⅱ—** **Emotion &Behaviour**

1. Feeling bad, useless, a burden to others. YES□ NO□2. Burst out laughing or Shouting. YES□ NO□3. Often groaning or lamenting in pain or discomfort. YES□ NO□

4. I had suicidal thoughts. YES□ NO□5. Act nervously. YES□ NO□6. Grab or rub the discomfort frequently. YES□ NO□7. Impatient with yourself and blame yourself. YES□ NO□8. No hope for the future. YES□ NO□

9. Suddenly panic. YES□ NO□

**Ⅲ— Body care& Movement**

1. Even with help, it's difficult to move, like getting in a car, taking a shower. YES□ NO□2. I need to hold onto things and be helped to get on or off a bed or chair. YES□ NO□3. Stand only for a short time. YES□ NO□4. Unable to maintain balance by my own. YES□ NO□5. Limited movement of hands or fingers. YES□ NO□6. Only with help can I stand up. YES□ NO□7. Kneel and bend only when holding onto other things. YES□ NO□8. Always in a restricted position. YES□ NO□9. Difficult to move body. YES□ NO□10. Hold onto something, such as a cane, to get on or off a bed or chair. YES□ NO□11. Lying down most of the time. YES□ NO□12. Change my position often. YES□ NO□13. I need to hold onto something to move on the bed. YES□ NO□14. Bathing needs a little help. YES□ NO□15. I can't bathe at all, need help. YES□ NO□16. I can use the bedpan with help. YES□ NO□17. Difficulty in wearing shoes and socks. YES□ NO□18. Inability to control urine. YES□ NO□19. I can't button up my own clothes. YES□ NO□20. It takes a long time to get dressed and undressed. YES□ NO□21. Inability to control feces. YES□ NO□22. I can dress yourself, but slowly. YES□ NO□

23. I have to be helped to get dressed. YES□ NO□

**Ⅳ— Home Management**

1. Do chores only for a short time. YES□ NO□2. Chores is done much less now than it used to be. YES□ NO□3. Not doing any of the chores I used to do. YES□ NO□4. I used to tinker, but don't do it anymore. YES□ NO□ 5. I used to go shopping, but don't do it anymore. YES□ NO□6 I used to clean the house, but don't do it anymore. YES□ NO□7. Manual work is difficult for me now, such as tinkering. YES□ NO□8. I used to washing clothes, but don't do it anymore. YES□ NO□9. I can't do any heavy housework. YES□ NO□

10. I can't manage household affairs, like depositing or withdrawing money. YES□ NO□

**Ⅴ— Mobiltiy**

1. I can only move in one house. YES□ NO□2. I can only stay indoors. YES□ NO□3. I spend more time in bed than before. YES□ NO□4. I stay in bed most of the time. YES□ NO□5. I can't squeeze into the bus now. YES□ NO□6. I stay at home most of the time. YES□ NO□7. I only go to places with lounges nearby. YES□ NO□8. I can't go to downtown anymore. YES□ NO□9. I have to go home after stay outside for a while. YES□ NO□10. I need help when I walk in dark places. YES□ NO□

**Ⅵ— Social &Interaction**

1. Seldom go out to visit relatives and friends. YES□ NO□2. Not going out to visit relatives and friends at all. YES□ NO□3. Not interested in other people's business and unwilling to help. YES□ NO□4. Always be angry with people around me, shout at them and reply sharply. YES□ NO□5. Rarely shows kindness to people around me. YES□ NO□6. Seldom participate in social activities. YES□ NO□7. The time spent visiting relatives and friends has been shortened. YES□ NO□

8. Avoid others’ social visit. YES□ NO□9. Sexual function is diminished. YES□ NO□10. Pay attention to changes in my health. YES□ NO□11. Rarely talk to people around me. YES□ NO□12. Asking too much of others, asking them to do things for me, and telling them how to do.

YES□ NO□13. Spent most of time alone. YES□ NO□14. I can't get along with my family. YES□ NO□15. I often get angry with my family and beat and scold them. YES□ NO□16. Spend as little time with my family as possible. YES□ NO□17. Seldom care about my children. YES□ NO□18. I ignore my family. YES□ NO□19. I used to care about the home and the kids, but now I don't. YES□ NO□20. Not joking with my family as usual. YES□ NO□

**Ⅶ— Ambulation**

1. The walk is shorter than I used to be, and there are frequent stops to rest. YES□ NO□2. I can't go up and down the hill. YES□ NO□3. I need cane to go up the stairs and hold on to the rail. YES□ NO□4. I have to be supported up the stairs. YES□ NO□5. I need a wheelchair to get around. YES□ NO□6. I can't walk at all. YES□ NO□7. Wobbly walk and fall easily. YES□ NO□8. I had to be supported to walk. YES□ NO□9. I go up and down stairs more slowly than before, often stopping. YES□ NO□

10. I can't walk up or down stairs at all. YES□ NO□11. I have to hold the wall and use a cane to walk. YES□ NO□12. Walking more slowly than before. YES□ NO□

**Ⅷ— Alertness Behaviour**

1. Start several things at once without a clue. YES□ NO□

2. I am more likely than ever to have minor accidents, like falling while walking and bumping into things.   YES□ NO□3. Being slow in reacting to others saying and behavior. YES□ NO□

4. Do not finish what I start. YES□ NO□5. Difficulty thinking and solving problems, such as making plans, making decisions, learning new things.   YES□ NO□

6. Sometimes confused. Not sure of the time, direction, where you are, who is around.

YES□ NO□

7. Be forgetful, such as where things are and if the door locked. YES□ NO□

8. Inability to concentrate for long time. YES□ NO□

9. Make more mistakes than usual. YES□ NO□10. Difficult to engage in activities that require thinking and concentration. YES□ NO□

**Ⅸ-Communication**

1. Dysgraphia. YES□ NO□2. Use gestures to communicate with others mostly. YES□ NO□

3. My words were understood only by a few people who understood me well. YES□ NO□4. The volume of speech is often uncontrollable. YES□ NO□5. I can't write anything but my signature. YES□ NO□6. I can't talk until I'm close to people or looking at them. YES□ NO□7. Difficulty speaking, such as choking, stuttering, or talking unclearly. YES□ NO□8. It is difficult for others to understand me. YES□ NO□9. I can't speak clearly when I'm nervous. YES□ NO□

**X—Recreation & Pastime**

1. Time for hobbies or entertainment has been shortened. YES□ NO□

2. Fewer recreational outings. YES□ NO□

3. Time for non-active pastimes (e.g., reading, television, playing cards) has decreased.

YES□ NO□

4. I've given up non-active pastimes (e.g., reading, television, cards).   YES□ NO□

5. I'm engaged in more non-active recreation instead of active recreation. YES□ NO□

6. I spend less time in group activities than I used to. YES□ NO□

7. Daily physical activity (such as playing basketball) decreased. YES□ NO□

8. I was no longer engaged in my usual physical activities. YES□ NO□

**XI— Eating**

1. I eat less than before. YES□ NO□

2. I can eat by myself, but it must be specially made for me, or special utensils. YES□ NO□

3. I eat a special diet, such as soft diet, low salt, low fat, low sugar or balanced diet. YES□ NO□

4. I can only eat liquid diet, such as milk and soybean milk. YES□ NO□5. I'm fussy about what I eat. YES□ NO□6. I seldom drink beverages now. YES□ NO□7. Need help with meals, such as setting the table. YES□ NO□8. I have to be fed. YES□ NO□9. I had to rely on gastric tubes or fluids to keep me nourished. YES□ NO□

**PART 2：Family APGAR index**

1. When I have problems, I can get satisfactory help from my family

Often□ Some times□ Rarely□

Supplementary explanation：

2. I am very satisfied with the way my family discuss various things and share problems with me Often□ Some times□ Rarely□

Supplementary explanation：

3. My family accepts and supports me when I wish to engage in new activities or developments

Often□ Some times□ Rarely□

Supplementary explanation：

4. I am satisfied with the way my family shows concern and love for my emotions.

Often□ Some times□ Rarely□

Supplementary explanation：

5. I am satisfied with the way my family spends time with me

Often□ Some times□ Rarely□

Supplementary explanation：
